# Sheep in Species-Rich Temperate Grassland: Combining Behavioral Observations with Vegetation Characterization

**DOI:** 10.3390/ani10091471

**Published:** 2020-08-21

**Authors:** Stephen J.G. Hall, Robert G.H. Bunce, David R. Arney, Elis Vollmer

**Affiliations:** Estonian University of Life Sciences, Kreutzwaldi 5, 51014 Tartu, Estonia; robert.bunce@emu.ee (R.G.H.B.); David.Arney@emu.ee (D.R.A.); Elis.Vollmer@emu.ee (E.V.)

**Keywords:** feeding behavior, vegetation analysis, video recording, grazing, pasture

## Abstract

**Simple Summary:**

Grasslands cover much of the world, and numerous people depend on the livestock that graze them for their livelihoods. These areas must be properly managed as they are often ecologically fragile. Therefore, how the foraging animal interacts with its environment needs to be understood. These interactions have mostly been studied in highly productive intensively managed and improved grasslands, which typically have only a limited number of commercially developed plant varieties. Little is known about how animals interact with less intensively managed, species-rich grasslands which are often of conservation significance because of their biodiversity. In this preliminary study, we have used video technology to investigate responses of sheep to the vegetation of unimproved grassland in Estonia. We classified the vegetation with a methodology that is standard in plant ecology but which has not been extensively applied in animal behavior. We also demonstrate the use of a novel procedure for quantifying foraging behavior. This combination of methodologies will enable the characterization of individual animal variations in these important behaviors, which could provide a basis for the rational design of sustainable grassland management systems.

**Abstract:**

Foraging behavior of livestock in species-rich, less intensively managed grassland communities will require different methodologies from those appropriate in floristically simple environments. In this pilot study on sheep in species-rich grassland in northern Estonia, foraging behavior and the plant species of the immediate area grazed by the sheep were registered by continually-recording Go-Pro cameras. From three days of observation of five sheep (706 animal-minutes), foraging behavior was documented. Five hundred and thirty-six still images were sampled, and a plant species list was compiled for each. Each plant species was assigned a score indicating its location, in the ecophysiological sense, on the main environmental gradient. The scores of the plant species present were averaged for each image. Thus, the fine structure of foraging behavior could be studied in parallel with the vegetation of the precise area being grazed. As expected, there was considerable individual variation, and we characterized foraging behavior by quantifying the patterns of interspersion of grazing and non-grazing behaviors. This combination of behavior recording and vegetation classification could enable a numerical analysis of the responses of grazing livestock to vegetation conditions.

## 1. Introduction

The trophic relationships between grasslands and herbivores are of clear ecological, evolutionary, and ethological interest [1,2]. Thirty years ago, the main practical application of scientific advances in these areas was to increase production from pastoral systems, the focus tending to be on pastures that had been seeded with economically valuable forage plants and managed accordingly. In recent years, appreciation of the productive potential of these systems has been paralleled by a concern for the conservation of traditional, unimproved (semi-natural) grassland habitats and their biodiversity, generally [3,4]. Linked with this is a growing interest in the sustainability of traditional husbandry systems in modern conditions, and in the potential for precision livestock farming in the context of extensification [5,6,7].

Reflecting the earlier production-oriented interest, experimental studies of the herbivore–pasture system have mostly been conducted in highly simplified environments often in conjunction with theoretical modeling [1]. Work was, in many cases, designed in such a way that fundamental scientific insights were achievable in such subject areas as motivation and optimality, but in the semi-natural grasslands that are of much current conservation interest, controlled experimentation [8] is seldom feasible. Here, behavior studies have mostly been of ranging behavior and activity patterns, usually by direct observation [9] and now often by movement recorders [10,11], but few studies have been made at finer scales. Understanding of the behavioral processes of the animal’s interaction with relatively complex plant communities has important contributions to make to the development of theory. As part of this process, we have investigated how the variability of animal and pasture can be codified and quantified.

The plant component has, at the local, or habitat, level, usually been investigated in relation to particular features of the vegetation by the spatial distribution of “major plant community types” [12] or by using such approaches as percentage cover of certain species [13], or relative biomass [14]. Ingested material can be assigned to species or to plant part by chemical or microscopical analysis [15,16] and under some circumstances, by visual observation of grazing animals [17,18]. The possibility of applying objective multivariate vegetation classification techniques [19,20] directly to the area actually being grazed by the animal, seems not to have been considered.

These techniques are based on the occurrence of species and do not involve the measurement of species biomass. Their relevance to the feeding ecology would, therefore, be questionable if diet selection were purely based on maximizing intake rate but it is clear that diets of herbivores, such as sheep, “contain a mixture of food items with apparent disregard for the intake rate each offers” [8] and, indeed, plant species richness itself has been shown experimentally to influence voluntary intake [21,22,23,24]. While the available biomass of forage is clearly important, the species richness of semi-natural or species-rich grassland could be another determinant of sheep foraging behavior, and this is the focus of our investigation.

Foraging behavior is a complex suite of phenotypes determined by genetics, environment, and learning [25]. The aspects most intensely studied have been the measurable behavior processes most closely linked to voluntary feed intake, which are time spent grazing and the dynamics of forage harvesting and processing. This has led to the recognition of the fundamental importance of bite mass in determining voluntary feed intake [26]. Insights such as these can contribute to theoretical modeling, which requires the definition of the currency to be optimized (for example, time or energy), the decision variables, and the constraints [1]. However, for modeling to have predictive value regarding the foraging behavior of livestock under conditions of extensive grazing, other aspects of behavioral science will be of particular relevance, such as behavioral heterogeneity or personality [27,28], and analysis of the relationships between and among bouts of grazing and of the behaviors that intersperse them [29].

Foraging herbivores, such as sheep, intersperse the behavior of harvesting vegetation with intervals of other behaviors, notably walking, standing still, or social behavior. Criteria for differentiating and classifying these behaviors vary considerably between studies [29]. Terminology varies as well, and in relation to grazing, multiple terms are in use, notably meal, bout, and feeding station, which are usually defined afresh in each study. The term “grazing” may be used explicitly for the harvesting of vegetation, as we have used it, or may describe a period during which acquisition of plant material is predominant, with episodes of locomotion. Consequently, a comparison between studies requires caution.

In this pilot study, we have investigated how the plant and animal components of semi-natural grasslands can be studied in parallel, using methodologies that have not yet been applied in a coordinated way in these habitats. These are, first, the recording of foraging using animal-mounted video technology, second, the characterization of the vegetation by objective methods, and third, the quantification of the effects of plant diversity on elements of foraging behavior. For the last, we have used an innovative behavior methodology that captures the overall temporal pattern of foraging. We have considered at two levels the relationships between vegetation type and foraging behavior: within the grazing bout (typically about 11–20 s), and over the entire foraging period (about 100 min in our study).

We emphasize that we have not used video recording to define which plants are actually being prehended, rather, we are seeking relationships between the species compositions of the area being foraged by the animal, and the structure of foraging behavior. Formally, we are proposing and testing the hypothesis that aspects of the structure of foraging behavior can be predicted from the vegetational character of the area being grazed.

## 2. Materials and Methods

### 2.1. Study Area and Animals

The study took place on semi-natural species-rich grassland of neutral soil acidity on a farm within Lahemaa National Park, in northern Estonia. The situation was unusual in several respects, principally in that the flock was permanently accompanied by at least four Maremmana guard dogs because of a perceived threat of predation, principally from bears and wolves.

Fieldwork took place on three days between 9 and 14 July 2018. The weather was warm and sunny, and there had been virtually no rainfall for some weeks. The pasture had been heavily grazed, as indicated by widespread, desiccated sheep droppings, and about half of the flock had been temporarily removed to grazing elsewhere. The field had been rotovated and harrowed about 13 years previously and used for hay and some crop production (details are lacking). All the species present were typical of infertile grasslands with a few indicators of old meadows. The presence of plants of damp ground, such as the moss *Rhytidiadelphus squarrosus* and sedges (*Carex* spp.), along with indicators of drier ground, such as *Achillea millefolium*, *Anthoxanthum odoratum,* and dead fine-leaved grass (*Festuca ovina*), indicated variation across the study area in soil moisture, while bare ground, sheep droppings, and plants tolerant of trampling showed that the sheep themselves acted as an environmental factor.

The study flock numbered approximately 50 ewes, of about 40 kg body weight, with their weaned lambs born during the previous year. Sheep were of the Estonian Native breed [30]. There was no evidence of lameness or parasite problems, or other pathologies, in the flock.

The pattern of flock activity was for the animals to be housed in a barn at night and to forage in an adjacent field for three or four hours in the morning. The guard dogs would then accompany the flock back to the barn. While some foraging took place near the barn during the afternoon, the main grazing period, typically of about 100 min, was in the early evening when the dogs would accompany the flock, across the field, to a 5 ha pasture about 250 m from the barn. It was during this later period that the observations were made.

Five mature, non-pregnant, non-lactating ewes were selected from among the larger-bodied sheep, based on the distinctiveness of coat color for ease of observation in associated behavioral studies (in preparation). Go-Pro Hero 5 Session camera (GoPro Inc., San Mateo, CA, USA) was fitted on each to a harness so as to have a field of view, while harvesting vegetation, from the forefeet to the lower forward extremity of the muzzle. Consequently, the actual plant material bitten by the sheep was only occasionally visible.

### 2.2. Video and Sound Monitoring

The GoPro cameras also recorded sound. This enabled counting of the bites that prehended vegetation (harvesting bites); sounds of chewing [31] were not discerned. These devices generated a maximum of six consecutive MP4 files, each of 17 min 42 s duration. The term “watch” is used here to designate the imagery and sound obtained on a single MP4 file, and the term “session” is applied to the set of up to six consecutive MP4 files obtained for a given sheep on a particular day. The total number of usable watches obtained was 49.

### 2.3. Behavior Data

Files were downloaded to a laptop, and behavior and interval bouts were then scored by a single researcher (S.J.G.H.), noting times of commencement and termination of bouts, and directly counting the number of bites and steps registered during each bout.

Data were analyzed for sheep individually. Bite and step rates were calculated. Following the definition of grazing time as “the time spent head down while searching or consuming” [32], we defined a “grazing” bout as a period during which the sheep has its muzzle down in the herbage layer and is harvesting vegetation; it begins with the first bite being taken and concludes when the animal raises its head above the herbage layer. Single bites, during locomotion, were frequently observed, but these have not been included in the analysis. By definition, each grazing bout was preceded and followed by an interval bout during which the animal might walk or run, or might remain stationary. The defining feature of the interval bout was that the animal’s head was out of the herbage layer.

A graphical method [33] was used to describe and quantify foraging behavior. A trajectory is plotted of cumulated time spent grazing (*x*-axis) against cumulated time spent in interval behavior (*y*-axis). The gradient of the best-fitting straight line is then calculated, forced through the origin (point 0,0). If the temporal structure of grazing and interval behaviors were regularly structured, with each grazing bout being followed by an interval bout of the same length, the fitted line would have a gradient of 1 and resemble a staircase with equal-sized treads and risers. Otherwise, the gradient will deviate from 1, a lower value indicating a predominance of grazing behavior. As a numerical descriptor of the structure of foraging behavior, this gradient has the advantage of behaving, when arcsin-transformed, as a normally distributed variable (unpublished data).

### 2.4. Vegetation Methods

Single frame images were sampled for vegetation analysis, balanced for grazing bout duration. For each watch, the following grazing bouts were sampled; the three longest, the three shortest, and four of approximately mean duration. The full sampling procedure, which was not possible for shorter bouts, was to examine three images within each sampled bout, that were closest to the mid-point of the bout, and the two that were midway between that point and either end of the bout. This yielded 536 images. Images from interval bouts (in which the behavior was almost always walking) were not analyzed.

The data for the complete set of images were coded as follows. While displayed on a laptop computer, presences of species were noted, and a list compiled for each image. Preliminary analysis indicated that 40 species were identifiable from the images and that between 1 and 10 (median 5) species were recorded in each image. One approach could have been to note the occurrence of plant species known to be palatable, and this would be appropriate in relatively species-poor grasslands, such as managed pastures. This would be an “agronomic” approach. While it may well be applicable in species-rich grasslands, we wished to investigate whether an “ecological” approach to characterization of vegetation would have predictive power in relation to grazing behavior.

The vegetation was characterized using the DECORANA procedure in Community Analysis Package version 5 [34,35]. In this well-known technique, multivariate methods are used to summarize patterns of correlation among plant species. As plants are correlated with each other, to varying extents, in their responses to environmental conditions, the overall correlation can be expressed in a correspondence analysis, and this enables plant communities to be defined objectively, as plant species of similar environmental requirements are located close together on the axes. Inspection of the axes and the locations of the plant species on them can indicate what factors explain the distribution of the species in the study area, as the ecophysiologies of these plants are well known. The main factors are likely to be soil moisture, nutrient status, soil acidity, and light regime. Because much of the variation is explained by the first axis, each plant species can, therefore, be defined, in ecophysiological terms, by its position, or score, on this axis. The scores of the plants visible in the image were averaged, so each image was, therefore, assigned a statistic, the mean first-axis score, which is indicative of the ecophysiological conditions in the area illustrated in the video image. Preliminary analysis showed that in our situation, a high first-axis score indicated relatively challenging ecophysiological conditions favoring plants that are relatively unproductive in agronomic terms.

Other multivariate statistics procedures would have been suitable, but we used DECORANA because we envisaged a more detailed phytosociological study of the plants, which we have not found necessary for the behavioral focus of this report.

### 2.5. Data Analysis

Statistical analysis and generation of graphics were by Microsoft Excel and by the *r* package [36,37]. Non-parametric tests were used when preliminary analysis (Shapiro-Wilks test) indicated the non-normality of data. Analysis of behavior data followed two approaches. First, grazing bouts and interval bouts were considered as distinct observations and the relationships among bout lengths, step rates, and bite rates investigated. Compliances of bout lengths to exponential distributions were tested with the *exp* function of R. If bout lengths fit such a distribution, the inference is that their termination occurs randomly and is considered to be out of the control of the animal [38]. Second, the patterns of grazing and interval bouts exhibited in each of the watches with at least seven grazing bouts (*n* = 43), was defined by their arcsin-transformed grazing-interval trajectories. Finally, the relationships between these grazing-interval patterns and the vegetation grazed were investigated.

Bout lengths were deduced from their start and finish times. For some bouts, this was not possible because the bout was in progress at the start or at the end of the respective MP4. file. For these bouts, bite and step rates could be obtained. This accounts for apparent inconsistences between some of the sample sizes tabulated here.

Considering the 5 sheep individually, a mean vegetation score was calculated for each grazing bout, and its correlations (Spearman, *r_s_*) calculated with the 3 behavioral measurements; grazing bout length, bite rate, and step rate during grazing.

## 3. Results

For behavior analysis, usable data were obtained from five individual sheep on three days, a total of 11 sessions (*n* = 49 watches). Of these 41 were of the full 17 min 42 s duration. Due principally to issues with harnesses, some watches were curtailed (six of 9–17 min, and two of 3–7 min). Recording times totaled 706 min 40 s.

### 3.1. Characteristics of Grazing and Interval Bouts

#### Bout Lengths

Complete grazing bouts totaled 1604, and complete interval bouts, 1542. The overall statistics are in Table 1. Sheep differed significantly in median lengths of grazing and interval bouts.

For all sheep, considered individually, distributions of bout lengths were found to fit exponential distributions. The proportions of variance explained by the exponential model were generally greater for grazing bouts than for interval bouts (Table 2).

For all sheep, combined, grazing bout length was negatively correlated with the lengths of the previous interval (−0.0845, *p* < 0.05) and the following interval (−0.141, *p* < 0.001). The step rate during the following interval was negatively correlated with grazing bout length (−0.127, *p* < 0.001). Bite rate was negatively correlated with grazing bout length (−0.519, *p* < 0.001). Numbers of observations contributing to these correlations (Spearman) varied between 1530 and 1587 as a result of censored or incomplete bouts having to be excluded.

### 3.2. Grazing-Interval Trajectories

**Figure 1:**
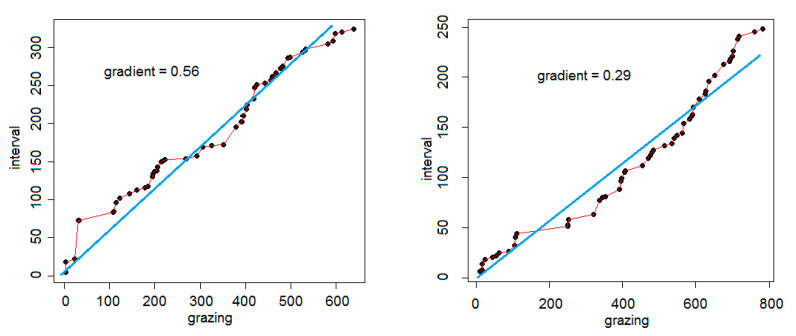
Grazing-interval trajectories for two specimen watches. For each, the cumulation, in seconds, of grazing bouts (*x*-axis) is plotted against that of interval bouts (*y*-axis). Total durations were 962 s (left-hand plot; 638 s grazing and 324 s interval) and 1030 s (right-hand plot; 782 s grazing and 248 s interval). The line of best fit, forced through the origin, is given with its gradient.

Grazing-interval trajectories were drawn for all watches of sufficient length (*n* = 46). Two examples of trajectories are given in Figure 1, showing the contrast between a watch dominated by interval behavior (gradient of 0.5599) and one by grazing behavior (0.2882). The grazing-interval trajectories showed compliance with normality when arcsin-transformed (Shapiro-Wilk test, *W* = 0.9628, *p* = 0.147, *n* = 46) and 27% of the variance was explained by differences among the five sheep (analysis of variance: *F* = 5.159, d.f. 4,41, *p* < 0.001). Untransformed arithmetic means were for individual sheep (n in brackets, total of 46 trajectories): B2, 0.53 (11); B4, 0.26 (9); W1, 0.43 (15); W2, 0.26 (4); W3, 0.35 (7).

### 3.3. Vegetation Data

Species noted included widespread species of grasslands of neutral soil acidity (*Agrostis capillaris*, *Anthoxanthum odoratum*) together with those of old meadows (*Alchemilla glabra*, *Chrysanthemum leucanthemum*), of infertile grassland (*Achillea millefolium*, *Campanula patula*), of impeded drainage (*Deschampsia caespitosa*), of dry relatively acid soils (*Festuca ovina, Rumex acetosella*), of moist/wet soils (three species of *Juncus*, *Viola palustris*), and low fertility soils (*Peltigera canina*, *Cladonia pyxidata*).

Ecological interpretation of the vegetation score was made based on the known ecologies of the plant species. In this study, a high score was found to be associated with lower soil fertility and greater soil moisture. Correspondingly, a low vegetation score implied greater fertility, associated with the occurrence of a small number of relatively fast-growing, competitive species which, in general, are of greater nutritional value.

### 3.4. Combining Vegetation and Behavioral Data

Of the 15 correlations between mean vegetation score for each grazing bout, and the 3 behavioral measurements (grazing bout length, bite rate, and step rate during grazing), 3 were significant, 2 of them relating to a single sheep.

**Figure 2:**
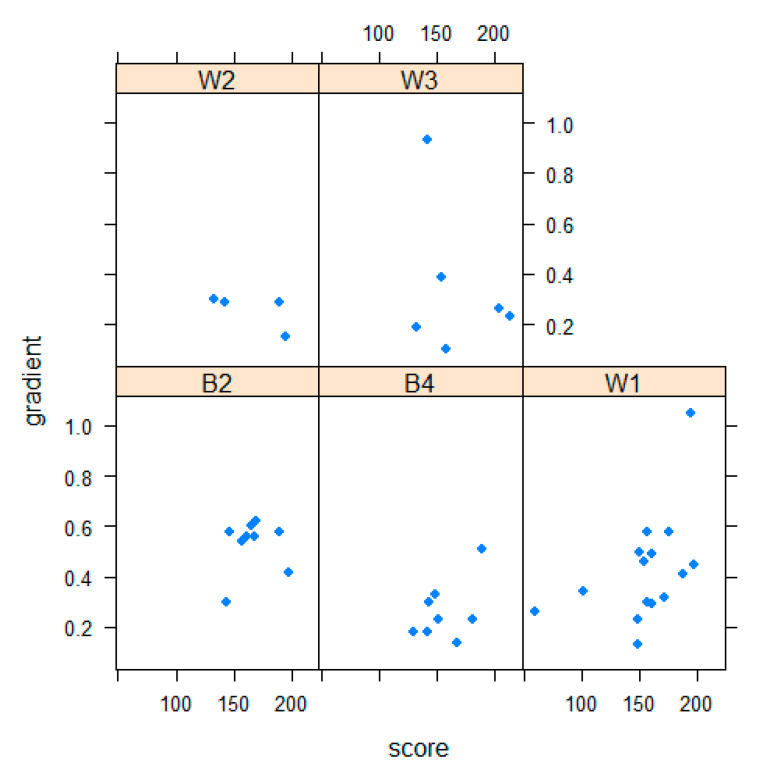
Plots, for individual sheep (identities respectively were W2, W3, B2, B4, W1) of gradient of the grazing-interval trajectory (*x*-axis) against mean vegetation score (*y*-axis) for each watch; 4 of the 46 points are masked in this diagram.

When the mean vegetation scores for grazing bouts were grouped according to the watches within which they were obtained, a composite vegetation score was obtained for each watch. Plotting this score against the gradient of the grazing-interval trajectory revealed the relationship between vegetation and the temporal structure of foraging behavior. When plots for each sheep were compared, differences among the individual behavioral responses to vegetation conditions became apparent (Figure 2). These were manifested by the distribution of points within each of the 5 plots. On the *x*-axis, higher values indicate a higher vegetation score, and on the *y*-axis, a tendency for foraging behavior to be oriented more towards interval and less towards grazing behavior.

## 4. Discussion

Improving the understanding of grazing in species-rich and semi-natural habitats could contribute to better management of these areas, which are of global economic and conservation significance [39]. The resilience of grasslands and rangelands generally is now acknowledged to be of fundamental economic and social importance [40]. Although there has been considerable progress in understanding the ecology of grazing systems “developments in … management … have been relatively slow and often empirical” [8]. Twenty years later, with the relevance of grazing management to conservation now more widely appreciated, it has been pointed out that “grazing is a major cause of failure to meet conservation targets for priority habitats in Europe” [41].

Work in these habitats has usually been in long-term and controlled, comparative studies focused on the consequences for plant diversity [42,43,44,45]. Less attention has been paid to the animal component. Our approach offers methodologies to supplement the experimental studies of how herbivores respond to the variability of vegetation, which has usually been in highly controlled situations. There, techniques have included such manipulations as patches being established, thus offering animals different plant morphologies [23,28,46,47,48,49].

The present study has been in a husbandry system, which is relatively unusual, but this is irrelevant to our conclusions, which primarily relate to the evaluation of these novel methodologies. The principal messages are that (1) Go-Pro technology can be applied, with only minor modifications principally in relation to harnessing, to sheep foraging ecology, (2) a standard vegetation classification based on multivariate statistics can be applicable to the behavioral ecology of animals, (3) this application can be facilitated by novel approaches to behavioral analysis and (4) the grazing-interval trajectory approach could provide an objective characterization of an individual animal’s foraging ecology.

### 4.1. Behavioral Components of Foraging Ecology

Considerable behavioral variation was found between individual sheep, as is common in herbivores [50]. Exponential models could be fitted to grazing and interval bout lengths. This implies that these bouts have a random element and are not under the complete control of the animal. The fit was better for grazing bouts implying that the random element in their lengths is greater than it is in interval bouts. The general pattern was that longer grazing bouts, which had lower bite rates, tended to be bracketed by shorter interval bouts, with lower step rates, while shorter grazing bouts, with higher bite rates, tend to be bracketed by longer interval bouts, with higher step rates. Further analysis would need to take account of individual variation and social effects in all these elements of behavior. The function of the interval bout in the ecology of herbivores has been studied extensively in relation to vigilance and social behaviors [51,52] and in relation to searching on a landscape scale, [53] but little is known about its role in the grazing of sheep at the pasture or field scale. We suggest that the grazing-interval trajectory model that we have used will enable characterization of foraging ecology, taking account of the interactions of grazing and interval behaviors.

Many previous studies in more intensively managed grasslands have interpreted foraging behavior as being composed of successive periods of harvesting small areas (feeding stations), which may then be aggregated for analysis into periods (bouts) during which harvesting is interspersed with short periods of non-harvesting behavior. Bouts may then be further aggregated into meals [32,54]). We could not apply this model because the sheep we observed did not show clear feeding station behavior. They interspersed harvesting bites with steps rather than remaining stationary during several successive bites. This has also been evident in other studies of sheep in extensive grasslands, such as in the Himalayas [53] and species-rich rangeland in France [55].

Bite rates in our study (median 96 bites min^−1^) were high, compared with those on sown grass swards (Soay sheep: body weight 16–38 kg, 56–67 bites min^−1^ [56]; Scottish Blackface sheep: 54 kg, 67–71 bites min^−1^ [57]). Presumably, in our study, the ratio of prehension to chewing bites [54] was higher than in studies on denser swards; bite mass may have been lower, requiring fewer masticatory jaw movements. In experimental conditions [57,58], bite rates and step rates do not show strong responses to vegetation conditions. Our finding of a lack of correlation of these rates with mean vegetation score implies this is also the case in species-rich conditions, and the behavioral response of sheep to the vegetation takes the form of altering the overall balance of grazing and interval behaviors in ways which show individual variability.

### 4.2. Variation in Behavioral Responses to Vegetation

These are preliminary results of a small-scale, pilot study, but there is evidently potential for combining objective vegetation characterization with novel behavioral approaches, so that differences among sheep in their responses to vegetation can be quantified. Sheep differed significantly in the gradients of their grazing-interval trajectories. Illustrating how these individual differences might interact with vegetation conditions, if the three sheep B2, B4, and W1 are compared (Figure 2), the first two are tending to graze in areas of middle to high vegetation score, but the structure of their behavior differs with B4 showing a rather lower value for grazing-interval gradient, indicating more intense grazing with less interval behavior. In contrast, W1 shows the whole range of both vegetation score and gradient. It is striking that this was also the sheep whose grazing bout length conformed most closely to an exponential distribution, such as would arise if the conclusion of a grazing bout is comparatively random. This would be the case if this animal had a particular tendency to be interrupted by others.

Foraging behavior is influenced by many physiological and environmental factors, and it is also an aspect of personality [50,59,60,61]. Elucidation of this complex phenotype requires it to be measured [62]. If it could be adequately expressed in a numerical form, there might be prospects for locating relevant genes, as has proved practicable for other behaviors [63].

## 5. Conclusions

The combination of well-established vegetation classification approaches with appropriate behavioral analysis may distinguish sheep—as individuals or as breeds—according to how their foraging style interacts with vegetation. A better understanding of this variability could contribute to the management, by grazing, of areas of conservation significance and the sustainability of rangeland grazing systems.

## Figures and Tables

**Table 1:** Grazing and non-grazing (interval) bouts. All variables were found to be significantly non-normal (Shapiro–Wilks test); between-sheep comparisons were by the Kruskal-Wallis test.

Behavior	Overall Median	Range of Medians Shown by Individual Sheep	Kruskal-Wallis Test Statistic (χ^2^)	*p*
Grazing bouts *n* = 1604				
Duration (s)	11	9–15	38.2	<0.001
Number of bites/bout	18	14–21.5	20.7	<0.001
Number of steps/bout	3	3–6	42.9	<0.001
Bites/minute	96	69.2–108.0	359.4	<0.001
Steps/minute	16.4	10.9–24.0	156.4	<0.001
Interval bouts *n* = 1542				
Duration (s)	4	4–5	15.9	<0.01
Number of steps/bout	4	3–5	45.9	<0.001
Steps/minute	68.6	60–80	49.2	<0.001

**Table 2:** Fit of bout lengths to exponential distributions, tabulated by individual sheep, with values of the *F* statistic (d.f. in brackets), and *r*^2^ (proportion of variance explained).

Identity of Sheep:	B2	B4	W1	W2	W3
Grazing bouts					
*F*	54.11 (1,66)	65.8 (1,78)	114.5 (1,76)	30.2 (1,51)	34.4 (1,59)
*p*	<0.001	<0.001	<0.001	<0.001	<0.001
*r^2^*	0.46	0.46	0.60	0.37	0.37
Interval bouts					
*F*	11.49 (1,37)	8.39 (1,24)	9.75 (1,35)	10.44 (1,19)	36.79 (1,20)
*p*	<0.01	<0.01	<0.01	<0.01	<0.001
*r^2^*	0.24	0.26	0.22	0.36	0.65
